# Acute high-altitude illness: risk factors, susceptibility prediction, and personalized prevention and treatment

**DOI:** 10.3389/fmed.2025.1735083

**Published:** 2026-01-12

**Authors:** Nan Jia, Chen Chen, Qian Chen, Junling Liu, Zherui Shen, Yuhan Liu, Caixia Pei, Yilan Wang, Demei Huang, Fei Wang, Yacong He, Zhenxing Wang

**Affiliations:** 1 Hospital of Chengdu University of Traditional Chinese Medicine, Chengdu, Sichuan, China; 2 School of Pharmacy, Chengdu University of Traditional Chinese Medicine, Chengdu, Sichuan, China

**Keywords:** AHAI, AMS, HACE, HAPE, high-altitude medicine, prediction, risk factors

## Abstract

**Background and objectives:**

Acute high-altitude illness (AHAI) comprises a spectrum of related conditions arising from exposure to high altitude, such as acute mountain sickness (AMS), high-altitude cerebral edema (HACE), and high-altitude pulmonary edema (HAPE). This study aimed to synthesize the existing evidence, delineate the risk factors and susceptibility predictors of AHAI, and outline personalized prevention and treatment strategies, as well as to identify key directions for future research.

**Methods:**

A systematic search of PubMed and Web of Science was conducted, such as clinical studies, systematic reviews, and authoritative guidelines published up to August 2025. No formal meta-analysis was performed; a narrative synthesis approach was employed to integrate the existing evidence.

**Results:**

Hypoxemia is the central pathophysiological driver of acute AHAI. Immutable host characteristics (age, sex, ethnicity, and genetic susceptibility) and modifiable comorbidities jointly influence baseline risk and disease trajectory. Current predictive approaches include hypoxic exercise testing and multifactorial risk scores; however, prospective, rigorously validated tools suitable for routine clinical use remain limited. Prevention and treatment strategies span non-pharmacological acclimatization, oxygen therapy, and pharmacologic interventions (e.g., acetazolamide, dexamethasone, calcium-channel blockers, and PDE5 inhibitors), with varying levels of evidence. Emerging therapies, such as traditional Chinese medicine, nanoparticle-based approaches, and psychological interventions, show promise. Management of AHAI should be individualized to accommodate patient-specific differences.

**Conclusion:**

Hypoxemia is the core pathophysiological driver of AHAI and is closely linked to the development of AMS, HACE, and HAPE. Individual responses to hypoxia show substantial heterogeneity, underscoring the need for personalized prevention and management strategies. Future study should develop more robust multi-parameter risk-prediction models and validate them prospectively across diverse populations and ascent contexts, and integrate wearable sensors, biomarkers, and novel drug-delivery systems into personalized interventions to enhance prevention and clinical outcomes of high-altitude exposure-related diseases.

## 1 Background

The prevention and management of acute high-altitude illness (AHAI) remain significant challenges in high-altitude environments. AHAI is a syndrome characterized by the rapid onset of central nervous system and respiratory symptoms following sudden exposure to hypoxic conditions at high altitude, primarily including acute mountain sickness (AMS), high-altitude cerebral edema (HACE), and high-altitude pulmonary edema (HAPE). Clinically, AHAI presents with a broad spectrum of neurological and respiratory symptoms. The incidence of AHAI is significantly influenced by altitude and individual acclimatization capacity, often leading to severe or even fatal complications within a short time frame (1, 2). Furthermore, due to individual variability, environmental factors, and heterogeneity of pathological changes, the prevention and control of AHAI remain challenging (3). This review provides a concise synthesis of the risk factors associated with AHAI and discusses the current strategies, evidence, and challenges in prediction, prevention, and treatment. It underscores the need to develop more precise, individualized intervention pathways to mitigate the burden of high-altitude exposure-related diseases.

## 2 Literature search and synthesis strategy

To ensure a comprehensive and balanced overview of current evidence, this review adopted a systematic literature search and selection process. Searches were conducted in electronic databases, such as PubMed and Web of Science. Given the relatively limited number of clinical studies focusing specifically on AHAI, no start-date restriction was applied; the search was updated until August 2025. The search strategy combined keywords (e.g., “acute high-altitude illness,” “acute mountain sickness,” “high-altitude pulmonary edema,” “high-altitude cerebral edema,” “risk factors,” “prevention,” and “treatment”) with corresponding Medical Subject Headings where applicable.

Inclusion criteria were as follows: (a) clinical research articles, systematic reviews, or authoritative consensus guidelines; (b) primary focus on the pathophysiology, risk factors, prevention, or clinical management of acute high-altitude illness in humans; and (c) publication in English or Chinese. Editorials and commentaries were excluded.

Titles and abstracts of retrieved records were screened for relevance, followed by full-text assessment of potentially eligible articles. In addition, reference lists of key articles were examined to identify further relevant literature. As this is a narrative review, no formal meta-analysis was performed.

## 3 Risk factors

**Figure 1:**
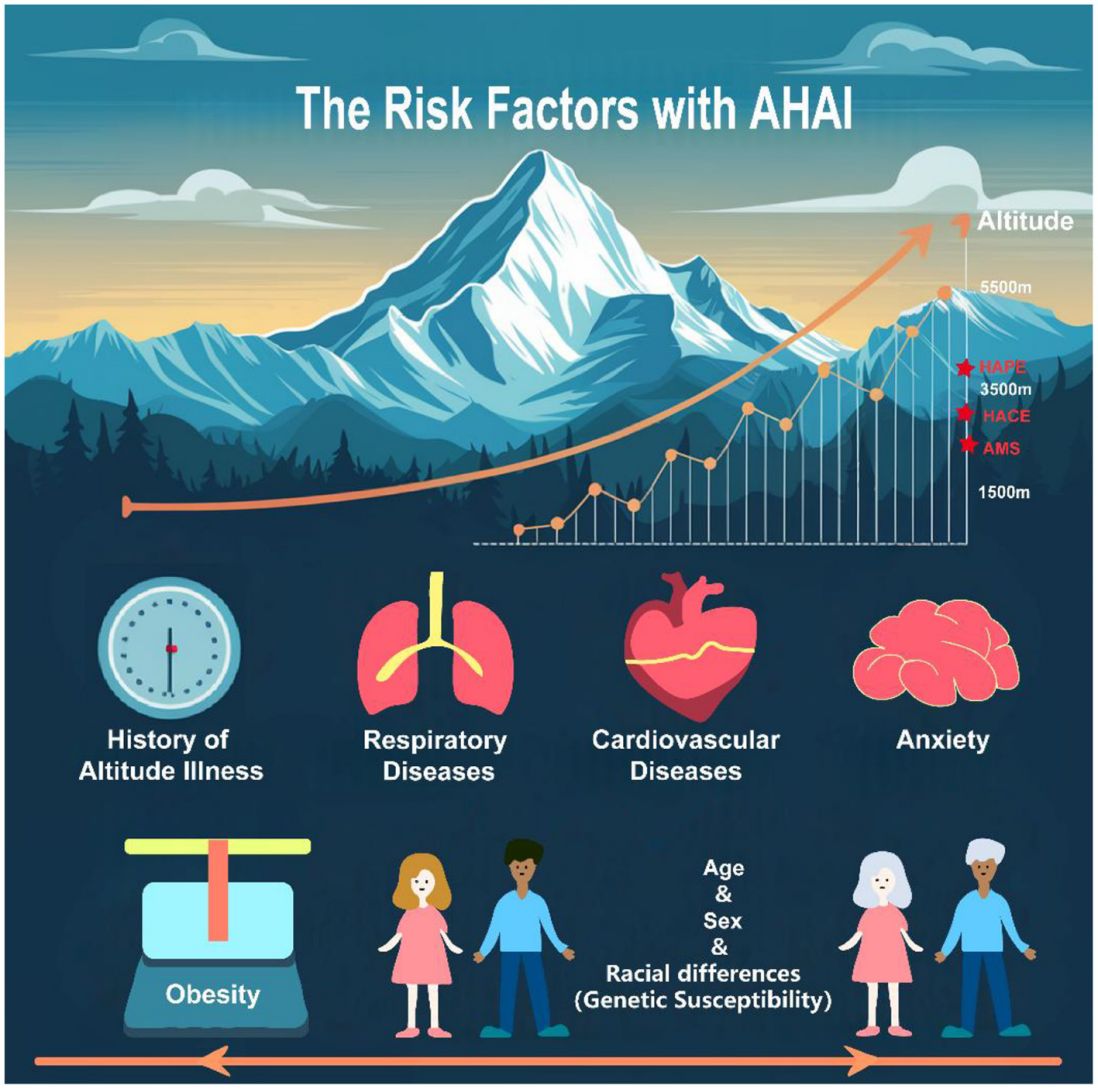
The risk factors with AHAI.

AHAI is not a single disease but a spectrum of related conditions. As AMS, HACE, and HAPE share common pathophysiological underpinnings, many risk factors may concurrently influence these three conditions; some factors have been shown to exert relatively disease-specific effects on the development of particular illnesses. Given that AMS diagnosis has a degree of subjectivity and that the incidences of HACE and HAPE are relatively low, investigations into the susceptibility mechanisms for the latter two remain limited. Commonly recognized consensus risk factors include first exposure to high altitude, rapid ascent, prolonged residence at low altitude, and a history of prior high-altitude illness (4, 5), which are briefly enumerated here. Hypoxemia is regarded as the shared final pathophysiological core of all risk factors and should therefore be a primary focus. Moreover, immutable individual characteristics such as age, sex, and ethnicity (and related genetic susceptibility) are important stratifiers of baseline risk for high-altitude illness. Simultaneously, as a class of modifiable comorbid risk factors, these should also be adequately considered in overall risk assessment and management. The risk factors associated with AHAI are depicted schematically in Figure 1.

### 3.1 Hypoxemia

Hypoxemia is an inevitable physiological alteration following exposure to high-altitude environments and constitutes the central pathological driver in the development and progression of AHAI. All other modifiable risk factors converge on hypoxemia as a pivotal node, influencing disease pathogenesis by exacerbating its severity, compromising compensatory responses to hypoxia, or amplifying hypoxia-induced tissue injury—thereby ultimately elevating disease risk. Through these multiple interrelated pathophysiological pathways, hypoxemia collectively contributes to the initiation of AHAI. Primarily, hypoxia directly induces oxidative stress and a systemic inflammatory response, promoting the release of pro-inflammatory mediators, such as interleukin-6 (IL-6) and tumor necrosis factor-alpha (TNF-α), thereby increasing vascular endothelial permeability (6, 7). In the brain, hypoxemia can lead to impaired cerebral autoregulation, resulting in abnormally increased cerebral blood flow and disruption of blood-brain barrier integrity, which constitutes a core mechanism for AMS and HACE (8). In the lungs, heterogeneous hypoxic pulmonary vasoconstriction elevates pulmonary arterial pressure, while concurrently, oxidative stress and inflammatory responses directly injure the pulmonary vascular endothelium; together, these processes promote the development of HAPE (9, 10). Further evidence indicates that the severity of hypoxemia is directly related to clinical manifestations. Across both cross-sectional survey studies and prospective observational studies, lower arterial oxygen saturation (SaO_2_) has been identified as an independent risk factor for high-altitude headache, and high-altitude headache is a defining clinical feature of AMS and HACE (11, 12).

### 3.2 Age

Age exerts a U-shaped, non-linear influence on the risk of AMS. Gonggalanzi et al. reported that age under 55 years is an independent risk factor for AMS, which may be attributed to an exaggerated hypoxic ventilatory response in younger individuals, leading to hyperventilation and resultant respiratory alkalosis (13). However, after the age of 70, the risk of AMS increases again, primarily due to a decline in cardiopulmonary reserve and a diminished compensatory capacity for hypoxia, leading to more severe and sustained tissue hypoxia (14). A meta-analysis further revealed heterogeneity in this relationship: among the included 12 studies, the prevalence of AMS decreased with advancing age; 11 studies reported no significant association; and 3 studies indicated a higher mean age among AMS patients (15). Collectively, these findings demonstrate that the relationship between age and AMS is not simply linear; its influence is likely mediated indirectly through effects on hypoxic adaptation and physiological compensatory capacity. Although the precise mechanisms by which age functions as an independent risk factor require further elucidation, and the increasing number of older individuals traveling to high altitudes underscores that age remains a crucial, non-negligible element in individual risk assessment and health management.

### 3.3 Sex

Studies have shown that sex exhibits distinct risk patterns for AHAI. Murdoch's report demonstrated AMS prevalence rates of 88.6% in women compared to 69.0% in men (16); similarly, Modesti et al. reported AMS rates of 60% vs. 21.9%, respectively (17). Consistent with these findings, a meta-analysis confirmed that the prevalence of AMS is significantly higher in women than in men (18). Notably, multiple retrospective analyses of clinical data have found that HAPE predominantly occurs in men (19, 20). Furthermore, a literature review conducted by the UIAA Medical Commission, which included seven studies focusing on women HAPE, revealed a lower incidence of HAPE in women compared to men (21). However, a scoping review found no evidence to suggest an increased risk of AMS or HACE in women (22). These sex differences may result from the combined effects of physiological factors and behavioral differences rather than from a single pathogenic factor. Hormonal fluctuations across the menstrual cycle in women may modulate ventilatory drive and cerebral blood flow regulation (23), thereby influencing sensitivity to hypoxemia and symptomatic presentation. In contrast, men under hypoxic exposure often exhibit a more pronounced pulmonary vasoconstrictive response, leading to a more significant elevation in pulmonary arterial pressure and consequently increasing the risk of high-altitude pulmonary edema (24). The role of sex as a risk factor for acute high-altitude illness (AHAI) has not yet reached a definitive conclusion. Its influence on disease susceptibility is not governed by a single mechanism but rather results from the complex interplay between hypoxic physiological adaptation and the endocrine environment. Future research is necessary to clarify, within this integrated framework, the specific pathways through which sex-based differences affect disease susceptibility and their relative contributions.

### 3.4 Racial differences (genetic susceptibility)

Racial differences often reflect underlying differences in genetic background, which plays a crucial role in an individual's adaptation to high-altitude, hypoxic environments, and their corresponding pathological responses. Population genetic analyses have revealed that Tibetans have developed a unique hypoxia-adaptive phenotype due to strong positive selection on EPAS1, while Andeans rely on high-frequency EGLN1 mutations. Compared to lowland populations, these groups exhibit significantly lower incidence rates of AHAI (25, 26). Zhou et al. identified significant associations between different HSP70 genotypes (such as hsp70-2 B/B and hsp70-hom A/B, B/B) and susceptibility to acute high-altitude illness, while the hsp70-hom A/B genotype may confer some tolerance to AHAI (27). Current evidence suggests that the genetic basis of AHAI involves multiple genes and their variants, which regulate hypoxic response and vascular function, interacting with environmental factors to influence individual adaptation to high-altitude conditions and the risk of disease development. Recent studies have revealed significant interindividual and interpopulation variability in the genetic susceptibility to AHAI. Among these, the EPAS1 and EGLN1 genes are particularly prominent. Similarly, a cross-sectional study in a Han Chinese population found that variations in EPAS1 and VEGFA genes are closely linked to AMS-related symptoms and susceptibility, reinforcing the impact of specific genetic variants on high-altitude adaptation (28). Martin et al. reviewed existing evidence on the genetic susceptibility to AMS, highlighting that AMS is a complex disorder resulting from the interaction of multiple genes and environmental factors, with genetic susceptibility being a key determinant of disease occurrence (29). Li Yuhong's transcriptomic analysis of HAPE patients revealed dynamic expression changes of key genes, such as BNIP3L, VEGFA, ANGPTL4, and EGLN1, under hypoxic conditions, underscoring their important regulatory roles during the acute and recovery phases of the disease (30). It should be noted that the aforementioned genes and their variants do not yet encompass the full spectrum of genetic factors associated with AHAI. Some genes exhibit distinct specificity in AMS and HAPE, and due to the limited reproducibility of studies and the complex interplay between genetic and environmental factors, further in-depth research is required to elucidate the precise underlying mechanisms.

### 3.5 Comorbidities

The impact of comorbidities on susceptibility to acute high-altitude illness AHAI has become increasingly evident, a process whose central mechanism is rooted in the compromise of physiological reserve and compensatory capacity in response to hypoxemia.

Respiratory diseases directly impair basal gas exchange function, diminishing the body's compensatory capacity in high-altitude environments and thereby predisposing patients to severe hypoxemia. Fiore et al. reported that respiratory infections substantially increase the risk of high-altitude illnesses, especially HAPE (31). Pre-existing respiratory infections are considered important risk factors for HAPE, with lower respiratory tract infections further exacerbating the risk. Recent case reports aligned with earlier studies suggest that respiratory infections may accelerate the onset and progression of HAPE (25, 32, 33). This may be attributed to shared pathophysiological mechanisms between the two conditions, likely through exacerbating local inflammation and vascular permeability, thereby exerting a synergistic pathogenic effect with high-altitude hypoxia.

Chronic pulmonary conditions, such as chronic bronchitis, emphysema, and chronic obstructive pulmonary disease (COPD), also increase the risk of AHAI (26). In a clinical study conducted by Gong et al., in outpatients with chronic bronchitis and/or emphysema, 17 out of 22 experienced varying degrees of AMS symptoms—such as chest tightness, dyspnea, dizziness, and headache—during high altitude travel (34). COPD patients may experience breathlessness even during mild exertion at sea level, with symptoms typically worsening in high-altitude environments. A prospective cohort study indicated that severe exercise-induced hypoxemia and a blunted ventilatory response to hypoxia are independent predictors of severe altitude illness, further highlighting the elevated risk faced by COPD patients at high altitude (35).

Cardiovascular diseases and respiratory diseases frequently coexist, thereby collectively limiting the body's capacity for oxygen delivery and utilization. Simulated testing predicting the health impacts on COPD patients in high-altitude environments suggests that hypoxemia at high altitude may trigger or exacerbate myocardial ischemia, arrhythmias, and other cardiac conditions, thereby accelerating adverse altitude-related health events (36). Additionally, studies have demonstrated that high-altitude exposure can induce myocardial hypoxia and adverse cardiac events, such as secondary myocardial infarction, thereby increasing the risk of AHAI (37). Physical activity at high altitude is advised against for patients with unstable angina, recent myocardial infarction, acute coronary syndromes, or those who have recently undergone angioplasty with stenting or coronary artery bypass grafting (38).

Obesity is closely correlated with the incidence of AHAI. A prospective study showed that obese individuals have a significantly higher risk of developing AHAI at high altitude compared to those with normal weight (39). Furthermore, obese individuals at high altitude often exhibit more pronounced hypoxemia and respiratory dysfunction, which may be key factors in the pathogenesis of AHAI (40).

In addition, anxiety has been confirmed by multiple studies as an important risk factor for AHAI (41–43). The high-altitude environment itself constitutes a potent physiological and psychological stressor, which readily induces or exacerbates anxiety, sleep disturbances, and panic symptoms. These psychological responses are closely associated with the worsening of AHAI symptoms. Mechanistically, anxiety can activate the sympathetic nervous system, disrupt sleep architecture, and trigger hyperventilation or dysfunctional breathing patterns. This in turn impairs the body's homeostatic regulation and compensatory adaptation to hypoxemia, ultimately establishing a vicious cycle of “psychological stress → physiological dysregulation → symptom exacerbation.”

## 4 Prediction of AHAI susceptibility

Accurate prediction of susceptibility to AHAI is of critical importance for preventing its occurrence and for developing individualized protective strategies. Currently, predictive methods predominantly rely on comprehensive analyses of multiple clinical, environmental, and physiological factors to enhance the accuracy of identifying at-risk populations. Canouï-Poitrine et al. developed a scoring system based on 10 clinical, environmental, and physiological variables that accurately predicts the risk of severe high-altitude illness in sea-level residents visiting high-altitude areas (44). This score integrates prior medical history, ventilatory and cardiac responses to hypoxia during exercise, ascent rate, oxygen saturation decline during hypoxic exercise, history of migraine, geographical location, women sex, age under 46 years, and regular physical activity. Using multivariate logistic regression, two models were established based on the presence or absence of previous high-altitude illness history. The results demonstrated that this scoring system facilitates the identification of highly susceptible individuals, thereby improving prevention strategies for severe high-altitude illness among newcomers to high altitude. Furthermore, the study emphasized the value of evaluating and quantifying hypoxic exercise tests to further predict severe high-altitude illness risk, especially for individuals without prior high-altitude experience.

Several related studies also support the application of hypoxic exercise testing for predicting AHAI. Kammerer et al. observed that post-exercise cerebral oxygenation decreases under hypobaric hypoxia were significantly negatively correlated with Lake Louise Scores 24 h later, suggesting cerebral oxygenation as a potential physiological biomarker (45). Richalet et al. identified multiple physiological risk factors through hypoxic exercise testing, such as ventilatory response to hypoxia during exercise, SpO_2_ decline, and cardiac responses during hypoxic exercise, with the latter two showing statistical significance in subjects not treated with acetazolamide (5). Markus Tannheimer et al. described a simple testing method combining the lowest SaO_2_ measured during high-altitude uphill running with completion time, comparing these to symptom severity at Mont Blanc summit (4,808 m), finding significant correlations between time, SaO_2_, and symptom severity (46).

At the physiological biomarker level, Matthias Peter Hilty et al. found that high-altitude exposure leads to increased plasma levels of soluble urokinase plasminogen activator receptor (suPAR), with a lack of correlation between suPAR and IL-6, indicating the presence of leukocyte-mediated, cytokine-independent low-grade inflammation. The correlation between IL-6 and arterial oxygen partial pressure reflected a direct effect of hypoxia. Notably, pre-exposure plasma suPAR levels could predict susceptibility to HAPE (47).

Additionally, Lacey et al. validated the feasibility of using an electronic nose to analyze exhaled volatile organic compounds (VOC) in remote high-altitude environments. Based on the hypothesis that hypoxia is closely linked to inflammation, they found differences in exhaled VOC profiles between AMS-resistant and susceptible individuals. Using LLS ≥ 3 as the AMS diagnostic threshold, the electronic nose effectively identified individuals at high risk for AMS (48). Thundiyil et al. demonstrated that an end-tidal CO_2_ (ETCO2) ≤ 22 mmHg had 100% sensitivity and 60% specificity for predicting AMS (49). Physiological parameters such as heart rate, respiratory rate, and oxygen saturation have also been widely confirmed as predictors of AHAI occurrence and severity (50–53). However, it is important to note that many of these indicators are based on physiological changes occurring after arrival at high altitude, limiting their utility for early prevention guidance.

## 5 AMS, HAPE, and HACE: characteristics of onset and interrelationships

AMS diagnosis primarily relies on clinical symptoms such as headache, loss of appetite, nausea, vomiting, dizziness, fatigue, and insomnia (54). AMS can be triggered at relatively low altitudes (2,000–2,500 m) (55, 56), and its incidence demonstrates a classic dose–response relationship with increasing elevation (57). HAPE and HACE represent severe manifestations of AHAI and can be regarded as progressive stages within the spectrum of the disease. Although both are often linked with AMS, they may also occur independently without preceding typical AMS symptoms. For rapidly ascending, unacclimatized individuals, the risk of HAPE increases substantially at altitudes above 3,500 m, and it primarily manifests as acute respiratory failure and pulmonary edema (58). It is a rare but life-threatening condition that is primarily observed after rapid ascent to altitudes exceeding 2,500 m (54). HACE is characterized by core neurological manifestations, such as ataxia, severe headache, vomiting, visual disturbances, and altered consciousness, with severe cases potentially progressing to coma.

The incidence rates of these three conditions differ significantly. AMS prevalence varies widely depending on population and altitude, ranging from approximately 20%−75% (2). Wang et al. reported that the incidence of related headaches reached 74.84%, while severe AMS accounted for about 23.7% (59). In contrast, the incidence of HAPE is lower. Burtscher et al. demonstrated through cohort studies that the incidence generally ranges between 1.7% and 6% (60). HACE is comparatively rare. Kai Schommer et al. conducted a meta-analysis indicating that the incidence of HACE is approximately 0.5% to 1%, often accompanied by severe symptoms of AMS (61).

**Figure 2:**
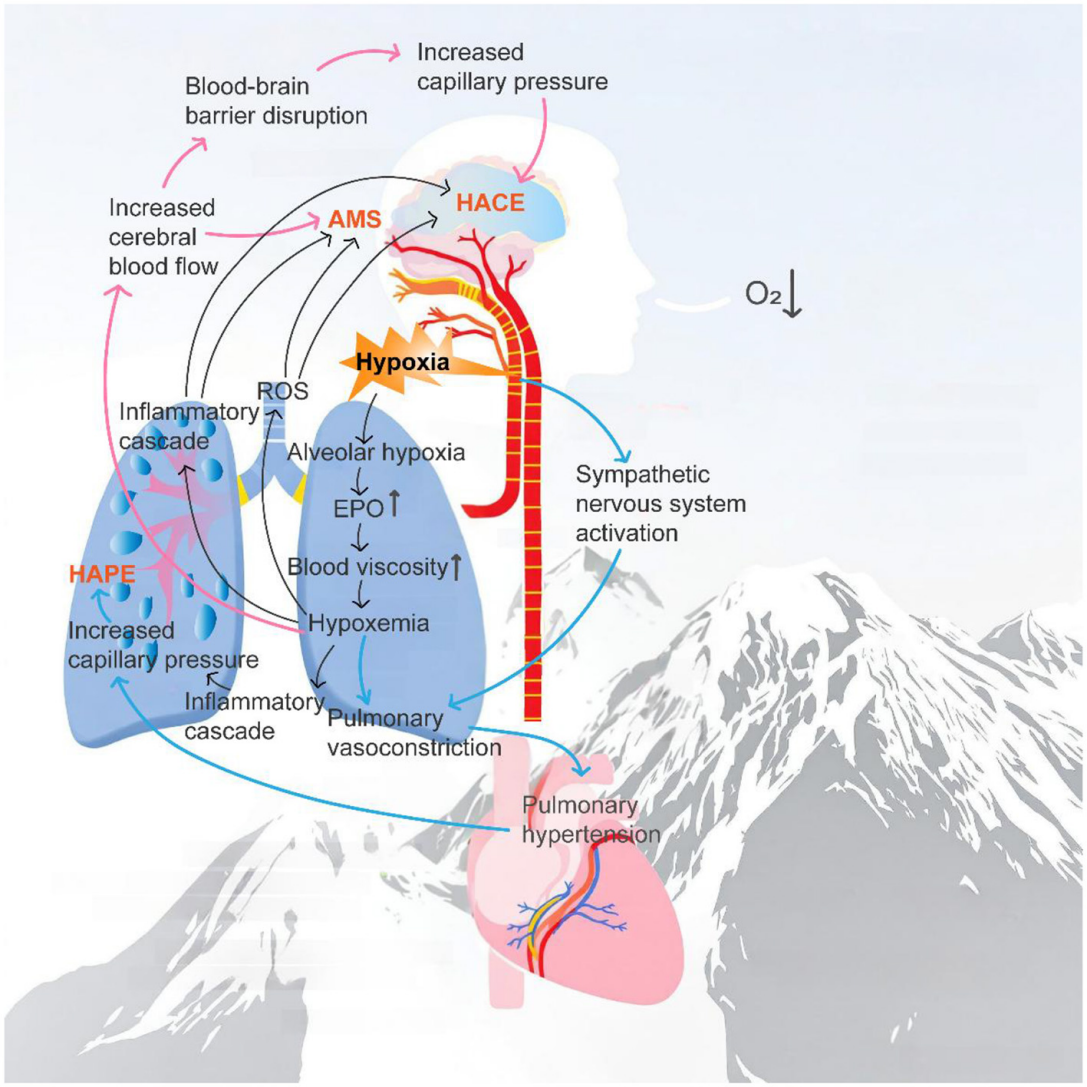
The pathogenic mechanisms of AMS, HAPE, and HACE and their interactions.

Although AMS, HAPE, and HACE present clinically as distinct conditions, they share a core pathophysiological mechanism initiated by hypoxemia (62). Under hypoxic stress, activation of the sympathetic nervous system triggers initial adaptive vascular responses in the cerebral and pulmonary circulations (63). In susceptible individuals, however, these compensatory responses may become maladaptive and progress—via common pathways involving the release of inflammatory mediators, enhanced oxidative stress, vascular endothelial dysfunction, and increased capillary permeability—ultimately leading to pulmonary or cerebral edema (59). While severe or progressive AMS is an important clinical precursor to HACE, not all cases of HACE are preceded by a typical AMS phase (64, 65). Hackett et al. reported that patients with HACE often deteriorate rapidly within 4 to 5 days after the onset of early AMS symptoms, presenting with neurological signs such as ataxia and altered consciousness (1). Clark et al. reviewed clinical cases and found that approximately 5% to 10% of severe HAPE patients also exhibited symptoms of HACE. Their study emphasized that HAPE and HACE frequently co-occur, suggesting a shared pathological basis involving increased capillary permeability and impaired oxygenation (66). Under hypoxic conditions, AMS typically presents as the earliest clinical response. Hypoxia stimulates the sympathetic nervous system, inducing vasoconstriction in cerebral and pulmonary vessels. Although this regulation aids short-term adaptation in severe hypoxia and HAPE, increased pulmonary capillary pressure exacerbates hypoxemia, triggering abnormal cerebral blood flow increases and blood–brain barrier disruption, ultimately leading to HACE (67–69). Figure 2 illustrates the pathogenic mechanisms of AMS, HAPE, and HACE and their interactions, outlining the pathological progression from initial hypoxic response to severe complications.

## 6 Prevention and treatment

### 6.1 Non-pharmacological prevention and treatment

For patients presenting with isolated headache or mild-to-moderate AMS, symptoms may be managed with rest, and they can safely remain at their current altitude, provided that further ascent is halted until symptoms fully resolve, physical exertion is limited, and adequate fluids are consumed to avoid dehydration (70). In cases with severe symptoms, immediate descent to a lower altitude is the most effective treatment method (71). A lower altitude environment provides a higher partial pressure of oxygen, which can rapidly improve tissue oxygenation and alleviate hypoxia-induced cerebral and pulmonary edema. Research indicates that rapid descent can significantly improve patient symptoms and reduce the risk of complications (24).

Oxygen therapy is the most fundamental and critical intervention in the management of AHAI, as it rapidly alleviates tissue hypoxia and effectively prevents further deterioration of the condition. Numerous clinical guidelines and studies have demonstrated that timely oxygen supplementation significantly reduces the risk of cerebral and pulmonary edema in patients with AMS, thereby improving oxygenation status (1, 72). Oxygen flow rates of 1–3 L/min have been widely used during sleep and ascent to maintain adequate blood oxygen levels, effectively mitigating symptoms of AMS and pulmonary edema (73). Recent studies have advocated the use of portable hyperbaric chambers as an on-site alternative therapy for acute high-altitude pulmonary and cerebral edema (74, 75). The literature indicates that hyperbaric chambers simulate reduced ambient pressure environments, significantly increasing arterial oxygen partial pressure and rapidly alleviating severe hypoxia and edema symptoms (76). This device plays a vital role when prompt evacuation to lower altitudes is not feasible. In recent years, portable oxygen concentrators have been increasingly utilized in high-altitude emergency care and long-term oxygen therapy due to their lightweight design and elimination of the need to transport oxygen cylinders. Research has shown that molecular sieve-based concentrators can deliver continuous medical-grade oxygen at altitudes up to 5,050 meters, markedly improving exercise capacity and symptoms of AHAI (77).

Progressive acclimatization, such as gradual ascent and staged high-altitude adaptation training, is widely recognized as an effective strategy for preventing AHAI. Extensive clinical studies and guidelines indicate that limiting daily altitude gain to no more than 300 meters, combined with adequate rest, significantly reduces the incidence of altitude illness (78). Additionally, avoiding strenuous physical exertion, maintaining proper hydration and electrolyte balance, and ensuring appropriate nutritional support are important adjunctive measures for the prevention of altitude-related illnesses (79).

### 6.2 Pharmacological prevention and treatment

Acetazolamide, as a cornerstone and first-line agent for the prevention and treatment of AHAI, holds an irreplaceable position in preventing AMS and HACE, as well as in treating AMS, constituting an essential component of comprehensive high-altitude acclimatization strategies (80). Its pharmacological mechanism involves the inhibition of carbonic anhydrase, which modulates acid–base balance and enhances ventilatory response in the respiratory center, thereby significantly improving oxygen saturation and alleviating hypoxic conditions (2). Multiple large-scale randomized controlled trials have consistently demonstrated that conventional dosing of acetazolamide (250 mg twice daily) reduces the incidence of AMS by approximately 50%, with good tolerability and mild, manageable adverse effects (1). A recent meta-analysis by Bartsch et al. further clarified the dose-dependent preventive effects of acetazolamide, providing robust evidence-based support for clinical dose optimization to improve efficacy and safety (81). Additionally, studies have indicated that combination therapy with acetazolamide and calcium channel blockers (such as nifedipine) may synergistically enhance gas exchange and therapeutic outcomes (82). Recent clinical trials have also explored the potential of combining acetazolamide with remote ischemic preconditioning, demonstrating a significant reduction in AMS incidence from 26.0% to 6.0%, highlighting a promising new avenue for multi-mechanistic synergistic protection (83). It is noteworthy that although acetazolamide is not a first-line treatment for HAPE, its ventilatory stimulation effects can indirectly alleviate hypoxemia and provide adjunctive protective benefits, underscoring its broad applicability and central role in the prevention and management of high-altitude illnesses.

Dexamethasone, as a cornerstone drug for the treatment of acute HACE, has been explicitly recommended as a first-line therapy in the 2024 Wilderness Medical Society (WMS) clinical practice guidelines; however, its efficacy in HAPE remains unconfirmed (72). The standard dosing regimen involves an initial 8 mg oral or intramuscular administration, followed by 4 mg every 6 h, which significantly alleviates neurological symptoms, rapidly reverses cerebral edema, and reduces mortality (84). Dexamethasone markedly suppresses the release of pro-inflammatory cytokines such as TNF-α, interleukin-1 beta (IL-1β), and IL-6, predominantly through inhibition of the nuclear factor kappa B and mito gen-activated protein kinase signaling pathways, thereby mitigating neuroinflammatory cell infiltration and inflammatory responses and protecting brain tissue from injury (85). Meta-analyses and randomized controlled trials suggest that dexamethasone dosage should be flexibly adjusted based on individual patient conditions to optimize therapeutic outcomes and minimize steroid-related side effects (86). In a double-blind study, combined treatment with dexamethasone and acetazolamide showed superior efficacy in improving AMS symptoms compared to monotherapy (87). However, this combination therapy has not yet been explicitly recommended by mainstream guidelines and requires further high-quality studies to validate its safety and effectiveness.

Nifedipine, a calcium channel blocker, has been traditionally used to alleviate pulmonary arterial hypertension associated with HAPE and improve pulmonary circulatory function. A systematic review by Luks et al. highlighted that nifedipine not only dilates pulmonary vessels by blocking calcium channels but also potentially mitigates hypoxia-induced endothelial inflammation and oxidative stress, thereby stabilizing the pulmonary capillary barrier and contributing to the prevention and treatment of HAPE (88). Evidence-based studies have further elucidated their multifaceted roles and optimized therapeutic strategies in the treatment of AHAI. Its use is supported by decades of clinical experience and evidence-based research. An early randomized controlled trial by Maggiorini et al. demonstrated that nifedipine significantly reduces the risk of HAPE in high-risk individuals, while also improving gas exchange and relieving symptoms (89). The trial employed an oral dose totaling 30 mg per day, showing notable efficacy with good tolerability. Subsequent meta-analyses have confirmed nifedipine as an effective agent for the prevention and treatment of HAPE, particularly in patients with a prior history of HAPE or pulmonary hypertension (90). A preclinical study reported by Zhu et al. demonstrated that high-altitude hypoxic conditions significantly inhibit the hepatic metabolism of nifedipine, resulting in increased plasma drug concentration and systemic exposure. These findings suggest that dosage adjustments may be necessary to avoid the risk of drug overdose (91).

Phosphodiesterase type 5 (PDE5) inhibitors, such as sildenafil and tadalafil, selectively inhibit phosphodiesterase-5 in pulmonary vascular smooth muscle, thereby increasing intracellular cyclic guanosine monophosphate (cGMP) levels. This promotes pulmonary vasodilation and reduces pulmonary arterial pressure, making them potential agents for the prevention and treatment of HAPE (92). This pulmonary vasodilatory effect helps improve oxygenation and clinical symptoms, which has garnered significant attention in high-altitude medicine in recent years. A randomized controlled trial by Bates et al. demonstrated that prophylactic oral sildenafil significantly lowered pulmonary artery systolic pressure, reduced the incidence of HAPE, and improved exercise capacity and oxygen saturation (93). Similarly, research by Maggiorini et al. further confirmed that sildenafil, through its pulmonary vasodilatory mechanism, can alleviate the severity of pulmonary edema and aid symptom improvement in affected patients (89). However, evidence regarding the efficacy of sildenafil for preventing AMS and treating HACE remains limited and inconsistent (94), indicating that its indication scope requires further clarification. The 2024 guidelines from the WMS recommend PDE-5 inhibitors as one of the therapeutic options for patients with clear indications, particularly in scenarios where immediate evacuation is not feasible or oxygen therapy is limited. However, the guidelines emphasize the importance of monitoring cardiopulmonary function to ensure patient safety (72). Currently, PDE-5 inhibitors are primarily used as adjunctive treatments in combination with oxygen therapy, evacuation, and other medications (such as nifedipine) to promote symptom relief and improve clinical outcomes.

**Figure 3:**
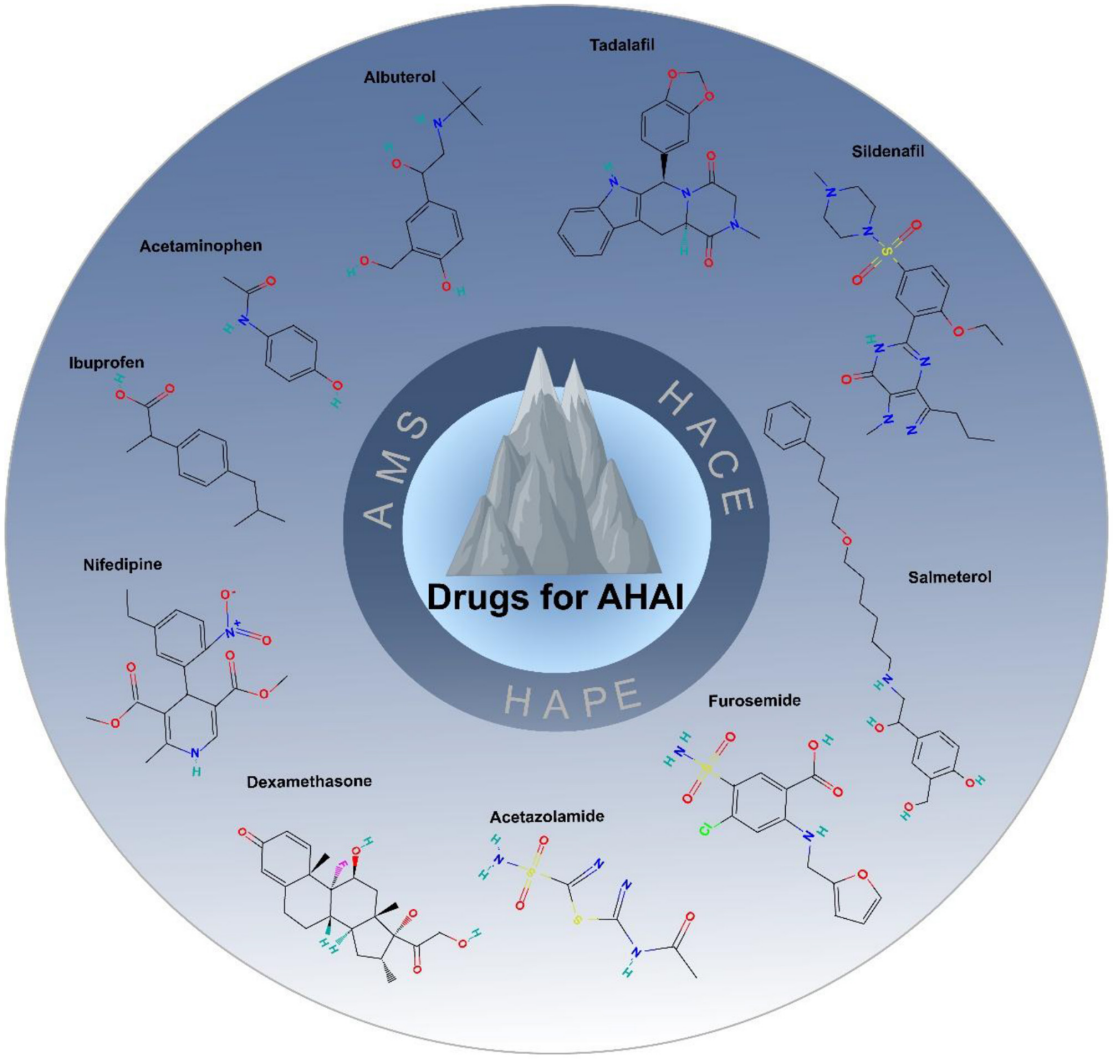
Drugs for AHAI.

In addition, studies have shown that ibuprofen effectively alleviates headaches associated with acute high-altitude illness but does not address the underlying pathophysiology, and should therefore be used as an adjunctive therapy, with caution regarding its potential gastrointestinal irritation and renal impairment (95). Acetaminophen serves as a relatively safe alternative for mild to moderate headaches, especially in patients at risk of gastrointestinal irritation, although hepatic function should be monitored (96). β2-adrenergic receptor agonists, such as salmeterol, have shown limited and inconsistent evidence in improving airway patency and oxygenation in patients with high-altitude pulmonary edema; their use is primarily adjunctive and requires cautious administration under expert medical supervision due to potential cardiovascular risks and altered pharmacokinetics in high-altitude environments (97). Diuretics, such as furosemide, may provide short-term symptom relief in pulmonary edema but lack sufficient evidence for routine prophylactic use, and excessive use may exacerbate dehydration and hemoconcentration, thereby increasing the risk of other high-altitude illnesses (98). Therefore, the use of diuretics should be carefully managed with strict biochemical monitoring and risk assessment. Figure 3 summarizes the main drugs used for the treatment of AHAI.

## 7 Novel therapeutic approaches

### 7.1 Traditional Chinese medicine (TCM)

In recent years, the role of TCM in the prevention and treatment of high-altitude illness has garnered increasing research attention. Its multi-component, multi-target holistic regulatory characteristics are considered promising for intervening in the intertwined pathological processes of hypoxia, inflammation, and oxidative stress under high-altitude conditions. Among single-herb interventions, *Rhodiola rosea* and *Ginkgo biloba* have been studied most extensively. Evidence suggests that *R. rosea* extract can elevate blood oxygen levels, alleviate clinical symptoms of AHAI, and demonstrate a favorable safety profile; however, its optimal dosage and efficacy require further validation through high-quality clinical trials (99). *G. biloba* extract has also shown potential (100) with study. Furthermore, TCM compound formulations leverage synergistic interactions among multiple components, offering potential advantages in anti-inflammatory, antioxidant, and microcirculation-improving effects. Related clinical research in this area is progressively emerging.

### 7.2 Nanoparticles (NPs)

NPs as novel drug delivery systems have demonstrated significant potential in the treatment of AHAI. NPs effectively protect encapsulated nucleic acid drugs from nuclease degradation, ensuring efficient expression or silencing of target genes in specific cells, thereby modulating pathophysiological processes at the molecular level and promoting disease amelioration. Studies have shown that nanoparticles, owing to their large surface area, high biocompatibility, and excellent degradability, serve as safe and efficient carriers for drug and gene delivery (101). Moreover, research indicates that many nanoparticles can function as magnetic resonance imaging (MRI) contrast agents, substantially enhancing MRI resolution and sensitivity, which provides an important tool for auxiliary diagnosis of AHAI. Li et al. demonstrated that multilamellar liposomal nanoparticles loaded with the calcium channel blocker alprenolol can rapidly release drugs into the circulatory system within 1 h after pulmonary arterial pressure (PAP) elevation, effectively reducing PAP and thereby treating HAPE (102). Additionally, preclinical studies have further shown that nanoparticles carrying anti-inflammatory drugs or RNA interference molecules can regulate the expression of inflammatory mediators, alleviate vascular permeability abnormalities, improve cerebrovascular function, and assist in mitigating related symptoms (103). It is noteworthy that nanoparticle formulations not only offer advantages such as simple preparation processes and storage stability but are also suitable for clinical application in the unique environment of high-altitude regions. The development of multimodal imaging nanoprobe technology provides strong technical support for the early diagnosis and precise treatment of AHAI.

### 7.3 Psychological intervention

AHAI patients often experience significant psychological stress and anxiety responses when exposed to high-altitude environments. This state is believed to potentially exacerbate the development and severity of high-altitude illness symptoms. Studies have shown that anxiety and stress at high altitude can impair the autonomic nervous system and endocrine responses, thereby reducing the body's adaptability to hypoxia and increasing the risk of AHAI onset (104). Furthermore, patients' fear of unfamiliar environments and potential health threats can further deteriorate their psychological state, negatively affecting clinical recovery. Gupta et al. demonstrated that relaxation training administered to individuals planning to ascend to high-altitude regions significantly reduced anxiety levels and alleviated mild to moderate symptoms of acute high-altitude reactions (105), highlighting the potential of psychological interventions as adjunctive measures to enhance high-altitude tolerance. Similarly, Wang et al. further emphasized that psychological stress impacts the autonomic nervous and endocrine systems, influencing adaptation to high-altitude environments, and underscored the supportive role of psychological interventions, such as relaxation training and cognitive guidance in mitigating AHAI symptoms and improving tolerance (59).

## 8 Management of AHAI

Management of AHAI in individuals exposed to high-altitude environments is a complex and highly challenging clinical task. Although public awareness of AHAI has increased, rapid transportation has become more accessible, wearable monitoring devices and portable hyperbaric chambers have been gradually adopted, high-altitude medical support systems have continued to improve, and the overall incidence of AHAI has not declined significantly. Meanwhile, as the number of people entering high-altitude regions grows and activities extend to higher and more remote areas, exposure to high-risk conditions has risen, contributing to a persistently elevated mortality rate among severe AHAI cases (106, 107).

Though recent advances have deepened our understanding of the pathophysiology and treatment modalities of AHAI, and multiple evidence-based studies have demonstrated the efficacy of certain interventions, there remains a lack of comprehensive risk assessment tools and standardized prevention and treatment protocols validated by large-scale randomized controlled trials. Common clinical management strategies include oxygen supplementation, pharmacological interventions, gradual acclimatization, and environmental adjustments, all of which have shown positive effects in symptom relief and reduction of AHAI incidence. Oxygen therapy is widely recognized as the cornerstone for correcting hypoxia. Pharmacological agents such as acetazolamide, dexamethasone, nifedipine, tadalafil, and ibuprofen have demonstrated preventive and therapeutic benefits in some clinical trials; however, optimal dosing regimens, combination therapies, and timing of administration remain controversial due to insufficient large-scale, high-quality randomized controlled evidence. Non-pharmacological interventions, such as staged ascent and pre-acclimatization training, have shown promise in facilitating physiological adaptation to high altitude but face limitations in rapid ascent scenarios and certain occupational exposures.

Until definitive AHAI prevention and treatment strategies are established, clinicians should tailor individualized management plans by integrating current clinical guidelines, the latest evidence-based research, and patient-specific factors. Although high-quality randomized controlled trials supporting TCM in effectively reducing AHAI incidence are lacking, existing clinical attempts suggest that TCM interventions are generally safe and free from significant adverse effects, making them suitable as adjunctive therapies (108). Furthermore, high-altitude exposure is often accompanied by psychological stress, anxiety, and sleep disturbances, which may exacerbate physiological symptoms or impair acclimatization. Psychological interventions such as counseling, stress management, and relaxation training can enhance patients' psychological resilience and improve overall adaptability.

With the rapid development of sensor technology and mobile health devices, portable wearable monitors have demonstrated broad potential in early detection of hypoxia and timely warning of altitude-related reactions, providing robust technical support for real-time monitoring and personalized management of AHAI (109, 110). Meanwhile, predictive models based on biomarkers, big data analytics, and genetic susceptibility assessments are increasingly refined, offering solid scientific foundations for precise screening of high-risk populations and customized pharmacological regimens (28). Novel drug delivery systems such as nanoparticles are also advancing, and by integrating these technologies with modern molecular targets and individual variability into multidimensional intervention strategies, more effective and precise prevention and treatment of AHAI are expected. Therefore, AHAI management should adopt a multidisciplinary, multimodal, comprehensive approach that combines pharmacological, non-pharmacological, and psychological interventions, supports technological applications, and considers individual differences to minimize the incidence and progression of AHAI.

## 9 Conclusion

AHAI onset and progression are substantially shaped by interindividual variability in hypoxic responses. Since hypoxia is the central pathophysiological driver of AHAI, effective prediction, prevention, and treatment of these conditions remain challenging. Individual differences (e.g., age, sex, and genetic susceptibility) and potential comorbidities may modulate AHAI risk. Current evidence indicates that predictive tools have limited clinical utility; future study should develop more robust multi-parameter risk prediction models and validate them prospectively across diverse populations and ascent contexts. Moreover, several alternative therapies, such as traditional Chinese medicine, nanomedicine, and psychological interventions, show promise but require additional clinical trials to establish safety and efficacy. To reduce the burden of altitude-related diseases, future management should integrate pharmacologic therapy, non-pharmacological interventions, and psychosocial support to deliver individualized, comprehensive treatment strategies tailored to the needs of different patients.
